# Alternative splicing regulation and its therapeutic potential in bladder cancer

**DOI:** 10.3389/fonc.2024.1402350

**Published:** 2024-07-26

**Authors:** Lina Li, Ting Jin, Liang Hu, Jin Ding

**Affiliations:** ^1^ College of Medicine, Jinhua University of Vocational Technology, Jinhua, Zhejiang, China; ^2^ Department of Gastroenterology, Affiliated Jinhua Hospital, Zhejiang University School of Medicine, Jinhua, Zhejiang, China; ^3^ Department of Urology, Affiliated Jinhua Hospital, Zhejiang University School of Medicine, Jinhua, Zhejiang, China

**Keywords:** alternative splicing, splicing factor irregularity, aberrant splicing events, bladder cancer, cancer therapy

## Abstract

Bladder cancer is one of the leading causes of mortality globally. The development of bladder cancer is closely associated with alternative splicing, which regulates human gene expression and enhances the diversity of functional proteins. Alternative splicing is a distinctive feature of bladder cancer, and as such, it may hold promise as a therapeutic target. This review aims to comprehensively discuss the current knowledge of alternative splicing in the context of bladder cancer. We review the process of alternative splicing and its regulation in bladder cancer. Moreover, we emphasize the significance of abnormal alternative splicing and splicing factor irregularities during bladder cancer progression. Finally, we explore the impact of alternative splicing on bladder cancer drug resistance and the potential of alternative splicing as a therapeutic target.

## Introduction

Bladder cancer is a prevalent form of cancer worldwide, and it ranks among the top 10 most commonly observed types of cancer (1). Due to the high recurrence rate of bladder cancer, it has a strong tendency to metastasize and has limited treatment options. As such, patients with bladder cancer suffer from a short survival time and a poor quality of life. Moreover, bladder cancer is often diagnosed in the advanced stages because of its insidious onset. For these reasons, bladder cancer is a significant healthcare burden.

Chemotherapy remains the primary treatment option for recurrent and metastatic urothelial carcinoma; however, the efficacy of chemotherapy is limited. In recent years, the advent of immunotherapy, particularly programmed cell death protein 1 (PD-1) and programmed-death ligand 1 (PD-L1) inhibitors, has presented new therapeutic opportunities for patients with bladder cancer (2). Consequently, identifying biomarkers that can assist in the diagnosis and treatment of bladder cancer is of critical importance.

**Figure 1:**
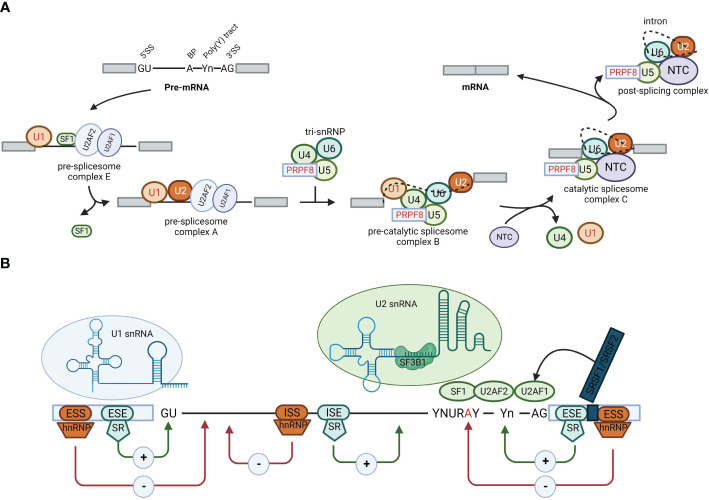
Alternative splicing. **(A)** Schematic representation of the stepwise assembly of the spliceosome from its small nuclear ribonucleoprotein (snRNP) components. U1 snRNP recognizes and binds to the 5′ splicing site, U2 small nuclear RNA auxiliary factor 2 (U2AF2) binds to the polypyrimidine poly (Y) tract, U2AF1 binds to the 3′ splicing site, and splicing factor 1 binds to the branch point, forming the pre-spliceosome complex E. Next, U2 snRNP replaces splicing factor 1 on the branch point to form pre-spliceosome complex **(A)** The tri-snRNP, consisting of U5, U4, and U6 snRNPs, joins complex A to form the pre-catalytic spliceosome complex **(B)** Thereafter, the U1 and U4 snRNPs leave, the U6 snRNP binds to the 5′ splicing site, and NTC is recruited so that the U6 snRNP and the U2 snRNP can pair, thereby generating the catalytic spliceosome complex C. Two subsequent transesterification reactions result in the creation of a post-splicing complex with intron exclusion and the formation of mature mRNA with interconnected exons. **(B)** Roles of cis-acting sequences and trans-acting factors in determining the splicing code for splice site selection. RNA-binding motif proteins, serine/arginine-rich family proteins (including SRSF2), and heterogeneous nuclear ribonucleoproteins bound to exonic or intronic regulatory elements can promote (+) or prevent (−) the recognition of the 5′ splicing site by the U1 snRNP or the 3′ splicing site by splicing factor 1, U2AF2, U2AF1, or U2 snRNP, thus affecting splice site choices and therefore alternative splicing decisions.

**Figure 2:**
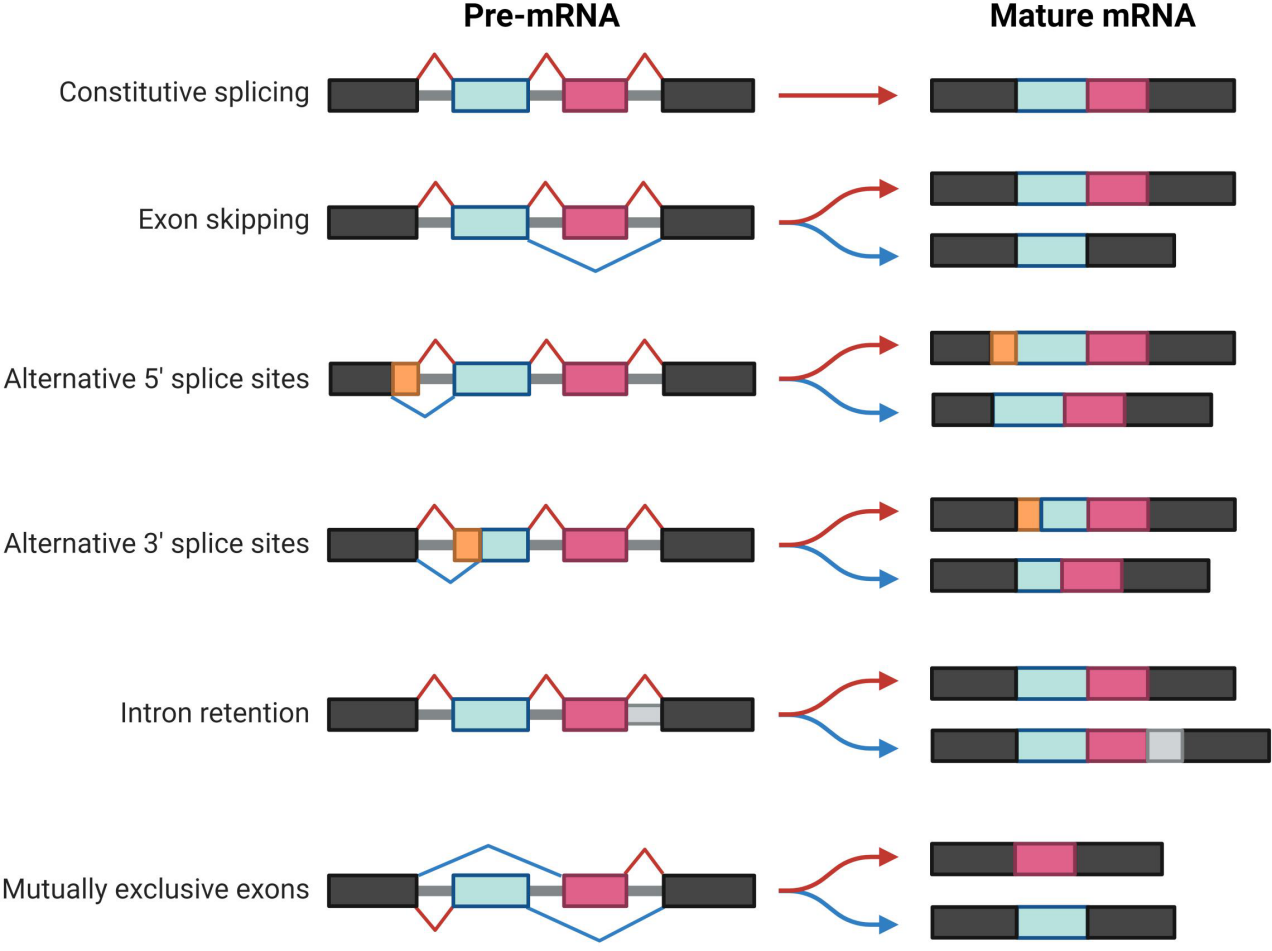
Summary of constitutive and alternative splicing events. The boxes represent exons, and the gray regions represent introns. The basic patterns of alternative splicing include exon skipping, alternative 5′ and 3′ splice sites, mutually exclusive exons, and intron retention.

Alternative splicing involves the conversion of pre-mRNA into mature mRNA. Through different splicing methods, a single gene can produce multiple distinct mature mRNAs, resulting in the production of distinct proteins (3, 4). Alternative splicing significantly contributes to the genetic and proteomic diversity of eukaryotes (5, 6) and is mainly catalyzed by spliceosomes (7). After identifying splicing signals (8) within pre-mRNA, spliceosomes engage with more than 300 proteins, referred to as splicing factors. Splicing factors bind to specific motifs or elements in the pre-mRNA, named splicing regulatory elements (SREs). Depending on their roles in splicing and their locations within the pre-mRNA, SREs can be divided into four subtypes: exonic splicing enhancers (ESEs), exonic splicing silencers (ESSs), intronic splicing enhancers (ISEs), and intronic splicing silencers (ISSs) (8–10) (**Figure 1**). These splicing complexes mediate the formation of multiple splice variants during different splicing events, such as exon skipping (SE), mutually exclusive exons (MXE), alternative 5′ splice sites (A5SS), alternative 3’ splice sites (A3SS), and intron retention (RI) (11) (**Figure 2**). Alternative splicing events occur in more than 90% of human genes and are precisely regulated within cells.

Alternative splicing frequently occurs in various disease states, including tumors, and it is thought to be involved in tumor progression. In fact, recent studies have revealed that alternative splicing plays a pivotal role in tumorigenesis, progression, invasion, metastasis, angiogenesis, and drug resistance (12–15). Multiple computational methods have been used to predict the roles of alternative splicing in tumors (16). Notably, several tumor-associated alternative splicing events have been identified in bladder cancer (17–19), indicating that a correlation may exist between alternative splicing and bladder cancer deterioration.

This review discusses the expression and function of splicing factors and the alternative splicing events in bladder cancer at cell lines, human or animal model level, and recent advancements in the therapeutic approaches targeting alternative splicing.

## Dysregulation of splicing-related proteins in bladder cancer

Alternative splicing is a remarkably intricate biological process that is meticulously regulated by the interplay between cis-elements (splicing signals and SREs) and trans-elements (spliceosomes and splicing factors) (**Figure 1**). These components give rise to a sophisticated regulatory network that operates in a coordinated and context-dependent manner.

Trans-elements are splicing-associated proteins that bind to specific RNA sequences or motifs. Splicing factors are a group of RNA binding factors that participate in the splicing of RNA precursors (20). Based on their functions, splicing factors are divided into two categories: small nuclear ribonucleoproteins (snRNPs) and non-snRNPs (21). After binding to pre-mRNAs, splicing factors either facilitate or hamper the interaction between spliceosomes and pre-mRNAs, suggesting their duality to either activate or inhibit alternative splicing. The evidence pertaining to protein changes associated with bladder cancer during RNA splicing is discussed herein.

### Serine/arginine-rich family

The serine/arginine-rich (SR) proteins, which include 12 evolutionarily conserved members (SRSF1–SRSF12), play a crucial role in protein synthesis. These proteins are characterized by an N-terminal RNA recognition motif and a C-terminal SR domain. SR proteins are involved in mRNA trafficking, peptide synthesis, and translation initiation complex formation (22) as well as pre-mRNA splicing and alternative splicing (23).

A previous study showed that the SR proteins are among the most dysregulated splicing factors in pan-cancers (24). Another study demonstrated that SRSF9 is upregulated in several cancers, including bladder cancer (25). Interestingly, patients with higher SRSF9 expression tended to have better outcomes after immunotherapy for bladder cancer (25). Furthermore, it has been shown that SRSF7 and SRSF10 expressions were associated with G3 tumors in non-muscle-invasive bladder cancer (26). More recently, Zeng et al. reported that SRSF1 promoted bladder cancer cell growth and migration by inducing exon 13 skipping of *CD46* (27). Another study reported that SRSF3 expression was significantly elevated in bladder cancer tissues. Moreover, *SRSF3* knockdown or removal of the SRSF3-binding motifs suppressed tumor cell growth by triggering A3SS activation within exon 18 of *ILF3* and promoting the skipping of exons 18 and 19, resulting in the production of interleukin enhancer-binding factor 3 (ILF3) isoforms 5 and 7 (28).

Taken together, this evidence demonstrates the potential of SR proteins as promoters in bladder cancer. However, the understanding of their role is still limited, and more in-depth research is needed.

### Heterogeneous nuclear ribonucleoprotein family

The heterogeneous nuclear ribonucleoprotein (hnRNP) family comprises a minimum of 20 members. These proteins possess common domains that bind to splicing silencers, in turn affecting splicing events, including constitutive splicing and alternative splicing, across the entire human genome (29, 30). In bladder cancer, hnRNPs participate in proliferation, apoptosis, angiogenesis, and invasion, contributing to cancer development and progression.

Protein hnRNP-A1 functions as an RNA-binding protein in mRNA splicing to regulate intron removal, exon joining, and mature mRNA formation. The multifaceted function of hnRNP-A1 in regulating the alternative splicing of numerous gene variants underscores its significance in bladder cancer—for instance, hnRNP-A1 mediates the c-Myc-enhanced pyruvate kinase (PK)M2/PKM1 isoform switch by selectively including exon 10 and excluding exon 9 during the alternative splicing of PKM pre-mRNA (31). Meanwhile, hnRNP-A1 interacts with the ESE element of CD46 mRNA, leading to exon 13 exclusion during splicing (32).

hnRNP-F is an RNA-binding protein that participates in various biological processes, including alternative splicing (33) and post-transcriptional modification (33, 34). Elevated hnRNP-F has been observed in bladder cancer, suggesting that it may play a role in tumorigenesis—for instance, Li et al. found that hnRNP-F was required for tumor growth and induced metastasis in bladder cancer. Specifically, hnRNP-F was significantly upregulated in bladder cancer tissues and was correlated with a poor prognosis in 103 patients with bladder cancer. When hnRNP-F was silenced or enhanced, Snail1 expression changed at both the mRNA and protein levels, implying that Snail1 may be a downstream target of hnRNP-F (35). Recent studies have also indicated a connection between hnRNP-F and phosphoinositide 3-kinase (PI3K)/protein kinase B (Akt) signaling. Inhibition of the PI3K/Akt pathway using the specific inhibitor LY294002 led to a significant decrease in hnRNP-F expression (36).

Epithelial splicing regulatory protein (ESRP)1 and ESRP2 are two RNA-binding proteins that belong to the hnRNP family. They have been shown to regulate the alternative splicing of fibroblast growth factor receptor 2 (FGFR2) pre-mRNA in epithelial tissues (37). The role of ESRP1 and ESRP2 in promoting splicing is reliant on the presence of ISE/ISS elements or the UGCAUG motif, which enhance FGFR2 transcripts from exon IIIc to exon IIIb splicing (38). Zhao et al. suggested that a decrease in ESRP1 or ESRP2 expression is related to lung metastasis in bladder cancer as it affects the splicing of FGFR2 and macrophage polarization (39). The hnRNP-F, hnRNP-H, and hnRNP-M proteins, which are closely related to ESRPs, also contribute to FGFR2 splicing regulation. Previous studies have shown that hnRNP-F, in association with hnRNP-M and hnRNP-H, suppresses FGFR2 exon IIIc, but it does not directly influence exon IIIb splicing (40, 41). However, no studies have addressed the FGFR2 splicing regulation of hnRNP-F, hnRNP-H, and hnRNP-M in bladder cancer.

According to previous studies, the complicated mechanisms underlying hnRNP-L alternative splicing regulation have been well studied in pan-cancers (42–45). Lv et al. (46) found that hnRNP-L expression was negatively correlated with the overall survival rate of 155 patients with bladder cancer. *HNRNPL* knockdown significantly inhibited cell proliferation and suppressed tumor development by promoting cell cycle arrest and apoptosis and inhibiting epithelial–mesenchymal transition. Moreover, it has been shown that hnRNP-L inhibits mitogen-activated protein kinase (MAPK) signaling. Therefore, the high expression of hnRNP-L in bladder cancer may lead to a poor prognosis and cancer development by suppressing intrinsic apoptotic signaling and promoting MAPK signaling (47).

In terms of cancer diagnosis and treatment, several studies have suggested that hnRNP-U may serve as a diagnostic marker for various cancers (48–51). An analysis of The Cancer Genome Atlas (TCGA) bladder cancer cohort also revealed a negative correlation between high hnRNP-U expression and patient survival, highlighting its detrimental impact—for instance, Shi et al. reported that hnRNP-U is highly expressed in bladder cancer, and inhibited hnRNP-U may improve chemotherapy sensitivity by enhancing cisplatin-induced apoptosis of bladder cancer cells (52). Similarly, hnRNP-K has been identified as a key factor associated with a poor prognosis in bladder cancer by influencing the proliferation and chemoresistance of bladder cancer cells (53).

Polypyrimidine tract-binding protein 1 (PTBP1) plays an essential role in splicing. It has demonstrated a positive association with tumor growth and is associated with a poor prognosis in various types of cancer (54–56). In bladder cancer, PTBP1 expression was found to be positively related to lymphatic metastasis, tumor stage, histological grade, and a poor patient prognosis in 104 patients with bladder cancer (57). Moreover, mechanistic studies have revealed that PTBP1 regulates massive splicing events in bladder cancer. Georgilis et al. found that PTBP1 regulates the development of inflammation-driven cancers by controlling exon 7 skipping of *EXOC7* (58, 59). Xie reported that PTBP1 promotes bladder cancer proliferation and metastasis by modulating the alternative mRNA splicing of MEIS2-L and PKM2 (57). Moreover, PTBP1 regulates the corresponding splicing events of numerous genes that are involved in tumor cell proliferation, growth, and metastasis, such as *TPM1*, *FAS*, *NUMB*, *MACF1*, *CD44*, *CTNND1*, and *ACTN1*(60).

### Spliceosome components

The spliceosome is a dynamic macromolecular complex that is responsible for pre-mRNA splicing, including internal intron removal and orderly exon connections (61–63). It is composed of five snRNPs (U1, U2, U4, U5, and U6) and a group of spliceosome-associated proteins. An snRNP comprises a small nuclear RNA (snRNA) molecule and a specific group of SNRP proteins (64). The SNRP proteins collectively form a structure that envelops the RNA molecule. The SNRP proteins share a conserved SNRP domain that regulates the step-by-step assembly of the snRNA (65, 66). Mutations in SNRP proteins can affect splicing patterns, potentially contributing to tumor development (67, 68).

The Sm proteins (B/B’, D1, D2, D3, E, F, and G) include seven members that form a ring-like structure that encloses the RNA molecule (64). Leili et al. (69) found that, of the 33 genes analyzed using the elastic net method, eight had an impact on the survival of 1,381 patients with bladder cancer. Among these, the expression of *SNRPE* was negatively correlated with survival time. However, to date, there are no new findings detailing the potential functional role of SNRP-E in bladder cancer.

Splicing factor 3A subunit 3 (SF3A3), which is encoded by *SF3A3*, is correlated with tumor stage and the prognosis of 49 patients with bladder cancer (70). Mechanistically, the transcription factor E2F6 interacts with KDM5C and binds to the promoter of *SF3A3*, leading to the demethylation of the GpC island associated with H3K4me2 and high SF3A3 expression.

Splicing factor 3B subunit 1 (SF3B1) is encoded by *SF3B1* and is a component of the U2 complex. It binds to the branch point nucleotide along with SF3B3 and PHF5A in the pre-catalytic spliceosome (71). Somatic mutations in *SF3B1* were initially identified by whole-exome sequencing in myelodysplastic syndrome (72), and they have since been identified in other hematological malignancies and solid tumors (73–79). Notably, Seiler et al. identified a specific *SF3B1* mutation (p.E902K), which was associated with A3SS events in bladder cancer samples (80). However, no subsequent studies have analyzed the aberrant splicing events caused by *SF3B1* mutations in bladder cancer.

### RNA-binding motif proteins

RNA-binding motif (RBM) proteins are a diverse family of proteins that contain an RNA recognition motif (RRM) domain that is involved in RNA binding. Several RBM proteins have been studied in the context of bladder cancer, including RBM3, RBM10, RBM24, RBM5, and RBMX. Among them, RBM3 has been identified as a marker of a poor prognosis in urothelial carcinoma (81, 82). Moreover, Seiler et al. reported a loss-of-function mutation in *RBM10*, which led to the induction of exon inclusion events (80). Other studies have shown that high RBM24 expression is associated with a poor prognosis in 32 patients with bladder cancer via the Runx1t1/TCF4/miR-625–5p positive feedback loop (83, 84). RBM5 is downregulated in bladder cancer, and its overexpression promotes apoptosis and inhibits tumor growth. Mechanistically, RBM5 is thought to promote tumor growth through a feedback loop involving miR-432–5p and β-catenin (85). The most thoroughly researched RBM protein in bladder cancer is RBMX, which is a ubiquitously expressed nuclear RNA-binding protein. Truncation mutations in *RBMX* have been observed in lung cancer through genome sequencing, suggesting its potential as a tumor suppressor (86). A previous study showed that RBMX competitively inhibits the binding of hnRNP-A1 to sequences flanking exon 9 of PKM through its RGG motif, resulting in a reduction in PKM2 expression and an increase in PKM1 expression. The attenuation of PKM2 expression suppresses tumorigenicity and bladder cancer progression. Additionally, RBMX inhibits aerobic glycolysis via hnRNP-A1-dependent PKM alternative splicing, and it counteracts the aggressive phenotype induced by PKM2 overexpression in bladder cancer cells (31).

### Others

Junction plakoglobin (JUP, γ-catenin) is a member of the Armadillo protein family (87). JUP forms complexes with adhesins and desmosomal adhesins, key components of the EXM (88). These desmosomal proteins mediate cell–cell interactions and signal transduction (89). JUP deletion leads to reduced cell contact, increased invasion, and bladder cancer cell metastasis (90). In line with this, the restoration of JUP expression could inhibit bladder cancer cell migration and tumor progression, and patients with bladder cancer with low JUP expression consistently exhibit poor survival rates (91, 92). JUP has also been reported as a key splicing factor that affects the metastasis and prognosis of other types of cancer (87). In bladder cancer, Huang et al. (93) showed that JUP is one of the splicing factors associated with overall survival-related alternative splicing events by the TCGA database.

Small nucleolar RNA (snoRNA) is a type of non-coding RNA that ranges in length from 60 to 300 nt. snoRNAs mainly originate from the introns of vertebrate host genes (94, 95). Initially considered to be transcriptional noise, snoRNAs are now known to regulate various biological processes (96–99). snoRNA dysregulation has been implicated in the development and progression of numerous diseases (100–104). Rui (105) investigated the correlation between five candidate snoRNAs (U49A, U3, SNORD19B, SNORD114–1, and SNORD113–9) and alternative splicing. Protein–protein interaction network analysis revealed that the related alternative splicing mRNAs clustered well. Moreover, Gene Ontology enrichment analysis revealed a significant enrichment of these snoRNAs related to EXM and focal adhesion. These findings suggest that snoRNAs may have an impact on alternative splicing events and potentially contribute to bladder cancer progression. In a case–control study involving 580 patients with bladder cancer and 1,101 control subjects, the single-nucleotide polymorphism rs978416 G>A in *RBFOX3* was potentially associated with bladder cancer predisposition in the Chinese population (106), indicating its potential as a novel biomarker of bladder cancer risk.

ALYREF, also known as RNA methyltransferase Aly/REF export factor, is an important “reader” protein that is located in the nucleus and that is involved in RNA processing and transport in cancer biology. It specifically recognizes and binds to m5C (methylated cytosine) sites within RNA molecules, thereby facilitating efficient RNA export from the nucleus to the cytoplasm. Notably, ALYREF interacts with various splicing factors, including SRSF3, PRPF3, and DHX16, suggesting its involvement in the intricate regulation of mRNA splicing (107). The function of ALYREF has been reported in the context of bladder cancer, where it leads to the occurrence of intron retention events in *RABL6* and *TK1*. Furthermore, an intriguing observation has been made regarding the m5C-dependent interplay between ALYREF and NOP2 Sun methyltransferase 2. Their cross-regulation promotes malignancy in urothelial bladder cancer, primarily by facilitating mRNA splicing and stabilization (108). These findings provide valuable insights into the underlying molecular mechanisms governing bladder cancer malignancy.

NONO, which is a member of the *Drosophila* behavior human splicing (DBHS) family, is an RNA-binding protein that is involved in diverse gene expression processes. Alongside its paralogs splicing factor proline and glutamine rich (SFPQ) and paraspeckle component 1 (PSPC1), NONO is predominantly localized to the nucleus and possesses two RRMs. These multifunctional proteins exert their regulatory roles by impacting various aspects of gene expression, such as transcriptional activation, inhibition, RNA splicing, stabilization, and export (109). NONO modulates the splicing events of *SETMAR* in bladder cancer by directly binding to its specific motifs, primarily through the RRM2 domain. Meanwhile, NONO interacts with the splicing factor SFPQ to further influence *SETMAR* alternative splicing. The activity of NONO is also associated with the suppression of bladder cancer cell metastasis (110). These findings illustrate the importance of NONO in bladder cancer progression and metastasis, shedding light on its potential as a therapeutic target in combating this disease.

Poly (U) binding specificity factor 60 (PUF60) participates in transcriptional regulation and splicing through direct binding to DNA or RNA (111). Although PUF60 is poorly studied in the context of cancer, its high expression has been detected in bladder cancer. Moreover, Long et al. identified a direct association between PUF60 and bladder cancer prognosis and aggressiveness by TCGA database (112). Mechanistically, this relationship was attributed to the transcriptional regulation of aurora kinase A (AURKA) by PUF60 on bladder cancer cell behavior (112). However, further research is needed to explore the precise mechanisms of PUF60 in bladder cancer and its potential as a therapeutic target.

Alternative splicing–splicing factor regulatory network analysis has identified several splicing factors, including pre-mRNA processing factor 39 (PRPF39), LUC7-like 3 pre-mRNA splicing factor (LUC7L), heat shock protein family A member 8 (HSPA8), and DEAD-box helicase 21 (DDX21), as potential biomarkers for bladder cancer (113). Moreover, eukaryotic translation initiation factor 3 subunit A (EIF3A), DDX21, SDE2, transportin 1 (TNPO1), and ring finger protein 40 (RNF40) are thought to mediate multiple alternative splicing events in bladder cancer, implying their essential roles in the development and progression of this disease (114). It is noteworthy that DDX21 has been associated with bladder cancer in several studies, but there are no mechanistic studies evaluating its involvement.

## Aberrant alternative splicing events of cancer-related genes associated with bladder cancer

It has been shown that there is usually a dysregulation of alternative splicing events in tumor, that is, a gene due to the occurrence of abnormal splicing leads to the large production of its pro-cancerous variants, which affects all aspects of the tumor. This section summarizes the aberrant splicing events that affect the TIME, prognosis, and disease progression of bladder cancer and summarizes them in **Table 1**.

**Table 1:** Summary of splicing signals in bladder cancer (BC).

SF	gene	Splicing event	Functional role	Molecular mechanism	RNA motif	Cell	Reference
hnRNP A1	PKM	Exon 10 inclusion and exon 9 exclusion	Inducing aggressive phenotype of the BC cells	PKM2 can increase glucose uptake and lactate production	Intronic UAGGGC sequences flanking exon 9	5637;T24	(31)
RBMX		Exon 10 inclusion and exon 9 exclusion	Attenuating the tumorigenicity and progression of BC	PKM1 can inhibit glucose uptake and lactate production	RBMX competitively inhibited the combination of the RGG motif in hnRNP A1 and the sequences flanking PKM exon 9	5637;T24	
NONO	SETMAR	Exon2 inclusion	Promoting metastasis	SETMAR-L mediated H3K27 trimethylation	CAGGCAGG sequence	UM-UC-3;T24	(110)
ESRP1	FGFR2	Shift splice form of FGFR2 IIIb to FGFR2 IIIc	Promoting proliferation, migration, and invasion	Affecting macrophage polarization	Binding to GU-rich sequences in ISE/ISS-3	T24; RT4	(39)
ESRP2							
PTBP1	MEIS2	MEIS2 exon 2 inclusion	Promoting migration and invasion	MEIS2-L overexpression rescued MMP9 expression	Binding sequence (UCUUC)	UM-UC-3;T24	(26, 57)
	PKM	PKM exon 10 inclusion and exon 9 exclusion	Promoting proliferation	PKM2 overexpression abolished downregulation of CCND1 expression	Not given	UM-UC-3;T24; RT4; RT112; EJ	
	EXOC7	Exon 7 skipping	Proto-cancer action	EXOC7 can regulate the pro-tumorigenic effects of the SASP	Binding PTBP1 RNA binding motifs on the upstream of exon 7	Senescent cells	(59)
	TPM1	Exon 6a inclusion and exon 6b exclusion	Cytoskeleton organization	Not given	Promoting exon inclusion when it binds in the downstream intron and exon skipping when it binds in the upstream intron	RT4, RT112; EJ	(26)
	FAS	Exon 6 skipping	Related to cell survival	Not given		RT4, RT112; EJ	
	NUMB	Exon 12 inclusion	Promoting proliferation	Not given		RT4, RT112; EJ	
	MACF1	Exon 99 inclusion	cytoskeleton organization			RT112	
	CD44	Inclusion of variable exons between v2 and v7	Interact with the extracellular matrix	Regulation of EMT markers		RT112	
	CTNND1	Exon 2 exclusion	Involved in cytoskeleton organization	Not given		RT4, RT112; EJ	
	ACTN1	Exon 19b inclusion and exon 19a exclusion	Involved in cytoskeleton organization	Not given		RT4; EJ	
SRSF1	CD46	Exon 13 exclusion	Promoting cell growth, migration, and tumorigenicity	CD46 regulate mRNA translation through an interaction with the translation machinery	Not given	EJ-1	(27, 32)
hnRNPA1					hnRNPA1 interacts with the ESE of CD46 exon 13		
PTBP1					Not given		
TIA1		Exon 13 inclusion					
TIAL1							
ZEB1	NIPBL	Facilitate back-splicing to promote the biogenesis of circNIPBL	Promoting migration and invasion	circNIPBL overexpression upregulates Wnt5a by targeting miR-16–2-3p	intron 145999–46005nt and intron 9618–624nt of NIPBL pre-mRNA	UM-UC-3	(115)
SRSF3	IL3	Exclusion/inclusion exon 18a/18b	Promoting proliferation	Enhancing cell cycle progression to the S and G2/M phase	AC-rich sequences in the ILF3 exon 18 in a size of 1,351 nt and exon 19 in a size of 362 nt	U2OS	(28)
ALYREF	RABL6	Intron retention	Promoting proliferation and migration	ALYREF and NSUN2 activate hypermethylated m5C oncogenic RNA through promoting splicing and maintaining stabilization	Binding to hypermethylated m5C site	T24	(108)
	TK1	Intron retention					
JUP	ITGB4	ITGB4–43,489-ES	Promoting bone metastasis and prognosis	Involving in the “glycosphingolipid biosynthesis ganglio series” pathway	Unknown	BLCA tissues	(93)

### Alternative splicing events associated with tumor immune microenvironment

**Figure 3:**
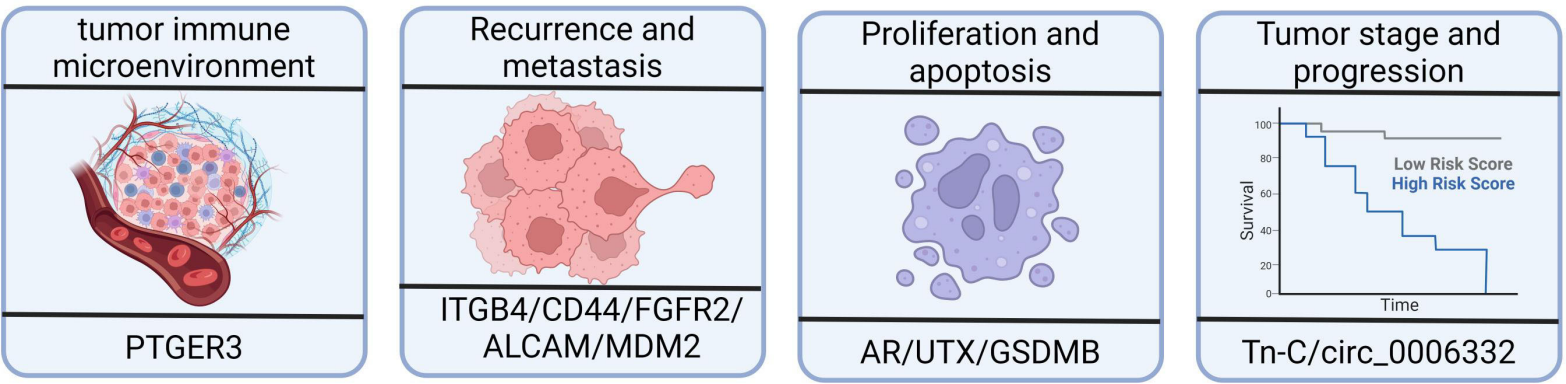
Effects of alternative splicing dysregulation on cancer progression. High prostaglandin E receptor 3 (PTGER3) expression may be associated with the immune response and the overall survival of patients with bladder cancer. Integrin subunit β4 (ITGB4) and CD44 are redistributed in bladder cancer tissues, impacting tumor migration and invasion. There is a higher proportion of shorter MDM2 alternative isoforms in bladder cancer patients with recurrence compared to patients without recurrence. ALCAM-Iso2 contributes to metastasis by increasing shedding and reducing cellular cohesion. The androgen receptor, UTX, and GSDMB isoforms play important roles in bladder cancer, including cell proliferation, viability, and cell death mechanisms. The expression of tenascin-C(L) variants is significantly increased in higher-stage and higher-grade tumors.

The tumor immune microenvironment (TIME) is composed of tumor cells, stromal cells, micro-vessels, interstitial fluid, and infiltrating cells, and it plays a crucial role in tumor occurrence, progression, and treatment (116–118). Evidence has suggested that the abnormal regulation of alternative splicing events has a significant impact on the composition and function of the TIME, ultimately influencing the prognosis of patients with bladder cancer. In bladder cancer, aberrant alternative splicing events have emerged as powerful biomarkers for evaluating the immune status, predicting prognosis, and identifying potential targeted therapies specifically in muscle-invasive bladder cancer (18, 85). A few notable studies have investigated the alternative splicing events of immune-related genes in bladder cancer—for example, the study of Li et al. (113) identified significant differences in the alternative splicing of *PTGER3* between bladder cancer tissues and healthy tissues using gene differential expression analysis (**Figure 3**). Patients with higher prostaglandin E receptor 3 (PTGER3) expression exhibited shorter overall survival but had higher immune checkpoint-related gene expression, more significant enrichment of immune signature-related genes, and a greater number of infiltrating immune cells compared with the low-expression group. Furthermore, Yu et al. confirmed the relationship between *TRMU* expression and immune cells and checkpoint genes using the TIMER database (18). These studies have provided insights into the potential roles of alternative splicing events in immune-related targets and their associations with immune activation and prognosis in bladder cancer. However, further research is still needed to explore the full extent of alternative splicing events in immune-related genes and their functional consequences in bladder cancer.

### Alternative splicing events associated with bladder cancer proliferation and apoptosis

Splicing defects in many vital genes are associated with tumor cell proliferation (119–121). The androgen receptor, which is a member of the steroid receptor family of transcription factors, is crucial for mediating androgen signaling, specifically testosterone and dihydrotestosterone signaling (122, 123). The study of Kimberley et al. (124) found that the majority of bladder cancer cells expressed different levels of low-molecular-weight (LMW) androgen receptor isoforms, while a small number expressed the full-length androgen receptor. Knockdown of total androgen receptor isoforms using silencing RNA reduced cell survival and triggered apoptosis, along with increasing the expression of nuclear androgen receptors. This finding indicates that LMW androgen receptor isoforms are normally present in bladder cancer cells and contribute to cell viability.

The C-terminal JmjC domain of UTX (ubiquitously transcribed tetratricopeptide repeat, X chromosome) is historically recognized for its histone demethylation function, contributing to its pathophysiological significance (125). In a previous study, multiple isoforms of UTX were identified in 5,637 cells. The three most abundant isoforms were lacking exon 14 (39% of UTX transcripts), exons 14 and 16 (27% of UTX transcripts), and the “long” isoform comprising all 30 exons (14% of UTX transcripts). Other identified isoforms included the isoforms lacking exon 16 (8%), exon 13 (6%), and exons 13 and 16 (4%), and there were several other isoforms with frequencies below 1%. The isoform lacking exon 14 exhibited weaker binding to chromatin, potentially due to its reduced nuclear abundance. The isoforms lacking exon 14 and exon 16 were unable to bind the polycomb repressive–deubiquitinase complex (PR-DUB) and mitotic deacetylase complex (MiDAC), which impaired the functional properties of the encoded protein (126).

Granzyme A, which is produced by killer lymphocytes, cleaves gasdermin B (GSDMB) and induces pyroptosis in tumor cells, leading to antitumor immune responses (**Figure 3**). Kong et al. discovered that different GSDMB isoforms have distinct functional properties. In their study, the N-terminal fragments of GSDMB isoforms 3 and 4, which are generated through cleavage, triggered pyroptosis. In contrast, isoforms 1, 2, and 5 failed to induce pyroptosis. The non-functional isoforms lacked a stable belt motif, which was either deleted or modified at exon 6. The belt motif facilitated the insertion of oligomeric GSDMB N-termini into the cell membrane. The expression of GSDMB3/4 isoforms, rather than GSDMB1/2, was commonly found to be upregulated in bladder cancers with better outcomes, indicating a protective role mediated by GSDMB3/4 in those tumors (127).

### Alternative splicing events associated with bladder cancer recurrence and metastasis

Patients with non-muscle invasive bladder cancer exhibit a high recurrence rate (60%) (128), with approximately 20% progressing to muscle-invasive bladder cancer (129). Recurrence or metastasis occurs in about half of patients with muscle-invasive bladder cancer who undergo radical cystectomy, leading to a low 5-year mortality rate of 50% (130). Prognosis is particularly poor in patients with locally progressive or recurrent muscle-invasive bladder cancer (131), highlighting the importance of predicting bladder cancer recurrence for effective management and treatment. Abnormal alternative splicing has been extensively studied in relation to bladder cancer recurrence, and the findings of these studies are summarized below.

Huang et al. (93) identified significant associations between specific alternative splicing events (SMOX-58,619-AP, INO80C-45,170-AP, and ITGB4–43,489-ES) and bone metastasis, splicing factors, and survival (**Figure 3**). In normal epithelial cells, integrin subunit β4 (ITGB4) binds to hemidesmosomes and promotes epithelial cell anchoring to the basement membrane. However, in tumors, ITGB4 is located at the front of the cell, which is rich in lamellae and filopodia, thereby enhancing tumor migration and invasion (132, 133). Another study showed that ITGB4 redistribution facilitated tumor migration and invasion, making it a valuable prognostic marker for bladder cancer (134). Moreover, JUP has been found to downregulate ITGB4–43,489-ES through the glycosphingolipid biosynthetic pathway, which is also associated with prognosis of bladder cancer (93).

CD44, a cell surface glycoprotein, has been extensively studied as a marker of bladder cancer aggressiveness and stemness. Alternative splicing and glycosylation generate multiple CD44 isoforms with distinct functional roles. Transcriptomics analysis has revealed significant heterogeneity among CD44 isoforms in 75 patients with bladder cancer, which is thought to be associated with tumor invasion and poor prognosis (135). Moreover, another study showed that a reduction in ESRP1 or ESRP2 promoted bladder cancer cell growth and lung metastasis by altering FGFR2 splicing and macrophage polarization (39).

MDM2, which is involved in the regulation of tumor suppressor p53, exhibits differential alternative splicing in patients with bladder cancer with or without recurrence. The expression of specific MDM2 exons is higher in patients with recurrence, indicating a higher proportion of shorter MDM2 alternative isoforms (136) (**Figure 3**).

Spliced variants of periostin, an EXM protein, have been detected in bladder cancer tissues and cell lines. In one study, wild-type periostin was reduced by downregulation or alternative splicing, particularly in isoforms lacking exon 18, and was strongly associated with bladder cancer progression. However, the lack of exon 18 did not suppress cancer cell invasiveness and metastasis (137). This indicates that the pro-metastatic function is achieved by aberrant alternative splicing to downregulate wild-type periostin expression.

ALCAM-Iso2, a splice variant of ALCAM, has been identified as a driver of metastasis. In one study, loss of the membrane-proximal region of ALCAM (exon 13) mediated by a novel matrix metallopeptidase 14-dependent membrane distal cleavage site increased shedding and decreased cellular cohesion, contributing to bladder tumor metastasis (138) (**Figure 3**).

Most studies have shown that the splicing abnormalities of some EXM-related proteins are closely associated with bladder cancer recurrence, providing valuable insights for mechanistic studies on relapse and metastasis.

### Alternative splicing events associated with tumor stage and progression

The primary transcript of the EXM protein, namely, tenascin-C (Tn-C), undergoes alternative splicing, resulting in the inclusion or omission of nine type III homology repeats. In bladder cancer, there is a significant increase in the expression of Tn-C(L) variants with a higher tumor stage and grade. Furthermore, the expression of Tn-C splicing variants differs depending on the tumor type, indicating differential regulation of Tn-C splicing in bladder cancer, which may contribute to its pathogenesis (139) (**Figure 3**).

Circ_0006332, which is derived from the splicing of *MYBL2* exons 8 and 9, has been implicated in bladder cancer progression. In a previous study, circ_0006332 knockdown inhibited the proliferation, colony formation, and invasiveness of bladder cancer cells. Moreover, E-cadherin was upregulated, while vimentin, cyclin B1, and P21 were downregulated, indicating Circ_0006332’s role in promoting epithelial–mesenchymal transition and cell cycle progression in bladder cancer (140).

## Targeted therapy of bladder cancer based on alternative splicing

Alternative splicing has emerged as a hallmark of cancer, and the development of therapeutic approaches targeting splicing holds great promise. Various tools have been developed to manipulate splicing, facilitating the identification of significant differential alternative splicing events associated with bladder cancer. These differential alternative splicing events, along with splicing factors, are thought to impact patients’ overall survival and chemotherapy drug sensitivity (141). Additionally, the prognostic signature based on alternative splicing is correlated with the response to common chemotherapeutic agents, indicating its potential as an indicator for treatment selection (140). Inhibitors of FGFR rearrangement, such as erdafitinib, infigratinib, and pemigatinib, have shown promise as targeted therapies against bladder cancer. Clinical trials are currently underway to explore the efficacy and safety profiles of these compounds in patients with bladder cancer with FGFR3 alterations (142). Perhaps increasing the sensitivity of chemotherapy drugs through targeted splicing is a relatively good way to achieve this.

Some drugs that may act by targeting alternative splicing are available. Enzalutamide, a second-generation therapeutic drug targeting the androgen receptor ligand-binding domain, has demonstrated effectiveness in inhibiting bladder cancer cells expressing the full-length androgen receptor (143, 144). However, the response to enzalutamide treatment appears to depend on the presence and localization of the full-length androgen receptor in the nuclear compartment rather than the absolute level of the full-length androgen receptor (124).

Targeted immunotherapy, particularly immune checkpoint inhibitor therapy, such as PD-1, PD-L1, and cytotoxic T-lymphocyte-associated protein 4 inhibitors, has demonstrated positive therapeutic effects in advanced bladder cancer (145–147). However, the effectiveness of immunotherapy varies among patients due to the heterogeneity of bladder cancer (148). Alternative splicing events not only regulate organ development, tissue identity acquisition, and tissue homeostasis but also play a significant role in tumor occurrence and development (149, 150). Furthermore, mutations that are tumor-specific to splicing factors have been identified as risk factors for tumor progression and maintenance (151), further emphasizing the potential of alternative splicing in immunotherapy (152).

Further research is warranted to unravel the complex mechanisms underlying splicing dysregulation in bladder cancer as well as to explore its therapeutic implications and advance precision medicine for this disease.

## Conclusions

RNA splicing is a crucial process that regulates gene expression and contributes to proteome diversity. Abnormal alternative splicing has been implicated in tumorigenesis in various cancers, including bladder cancer. This review summarizes splicing dysregulation in bladder cancer, particularly in terms of specific splicing variants and splicing factors, which have potential clinical value as diagnostic/prognostic biomarkers or therapeutic targets. Meanwhile, the impact of splicing dysregulation on TIME, disease progression, recurrence, and therapy resistance are emphasized. The review collates the findings of studies investigating splicing-related factors associated with bladder cancer, with a focus on splicing factors, particularly the hnRNP family of proteins. These factors are aberrantly expressed in bladder cancer and are associated with specific splicing events and clinical outcomes (**Table 1**). However, the underlying mechanisms remain largely unknown, necessitating further research to elucidate the oncogenic and tumor-suppressing functions of alternative splicing events and splicing factors in bladder cancer. Pre-clinical studies have illustrated the contribution of splicing events to treatment resistance and disease progression in bladder cancer, illustrating that these events could serve as therapeutic targets. Nonetheless, due to the lack of clinical implementation in patients with bladder cancer, the development of drugs targeting spliceosomes remains challenging. Future investigations should focus on unraveling the mechanisms underlying splicing abnormalities and the consequences of inhibiting these factors, thereby enabling the full potential of splicing-based therapies in bladder cancer to be recognized.

## Author contributions

LL: Writing – original draft, Writing – review & editing. TJ: Writing – review & editing, Writing – original draft. LH: Writing – review & editing. JD: Writing – review & editing.

## Glossary

**Table:**

PD-1	programmed cell death protein 1
PD-L1	programmed-death ligand 1
SREs	splicing regulatory elements
ESE	exonic splicing enhancer
ESS	exonic splicing silencer
ISE	intronic splicing enhancer
ISS	intronic splicing silencer
SE	exon skipping
MXE	mutually exclusive exons
A5SS	alternative 5′ splice sites
A3SS	alternative 3′ splice sites
RI	intron retention
snRNP	small nuclear ribonucleoprotein particle
SR	serine/arginine-rich
hnRNPs	heterogeneous nuclear ribonucleoproteins
hnRNP A1	heterogeneous nuclear ribonucleoprotein A1
hnRNPF	heterogeneous nuclear ribonucleoprotein F
PI3K	phosphoinositide 3-kinase
AKT	protein kinase B
FOXO1	Forkhead box O1
ESRP1	epithelial splicing-regulatory protein 1
ESRP2	epithelial splicing-regulatory protein 2
hnRNP-L	heterogeneous nuclear ribonucleoprotein L
LSCC	lung squamous cell carcinoma
PTBP1	polypyrimidine tract-binding protein 1
snRNA	small nuclear RNA
RBM	RNA-binding motif
RRM	RNA recognition motif
PKM	pyruvate kinase
JUP	junction plakoglobin
snoRNA	small nucleolar RNA
DBHS	*Drosophila* behavior human splicing
PUF60	poly (U) binding specificity factor 60
TIME	tumor immune microenvironment
UTX	ubiquitously transcribed tetratricopeptide repeat
GSDMB	gasdermin B
Tn-C	tenascin-C
